# Hybrid repair for Kommerell’s diverticulum and right aortic arch with aberrant right vertebral artery

**DOI:** 10.20407/fmj.2020-016

**Published:** 2021-03-20

**Authors:** Mika Noda, Hiroshi Ishikawa, Yoshiyuki Takami, Yusuke Sakurai, Kentaro Amano, Kiyotoshi Akita, Tatsuo Banno, Ryoichi Kato, Yasushi Takagi

**Affiliations:** 1 Department of Cardiovascular Surgery, Fujita Health University, School of Medicine, Toyoake, Aichi, Japan; 2 Department of Radiology, Fujita Health University, School of Medicine, Toyoake, Aichi, Japan

**Keywords:** Kommerell’s diverticulum, Right-sided aortic arch, Aberrant right vertebral artery

## Abstract

Kommerell’s diverticulum (KD) is a rare aneurysm of the origin of an aberrant subclavian artery. Hybrid aortic arch repair for KD is being performed more often. We report hybrid arch repair for KD in a 63-year-old man with a right aortic arch and aberrant right vertebral artery, an extremely rare variant. We performed total arch replacement to completely reconstruct the five cervical arteries with elephant trunk to create an adequate landing zone, followed by second-stage endovascular stent-grafting from the ascending aorta to the proximal descending aorta.

## Introduction

Kommerell’s diverticulum (KD) is a rare aneurysm of the origin of an aberrant subclavian artery from the descending aorta, with a prevalence of 0.04%–0.4%.^1^ KD exists with a left or right aortic arch as a remnant of the fourth primitive dorsal arch. Although KD appears as a pouch, measuring its diameter is difficult. A cross-sectional diameter from the opposite aortic wall to the tip of the diverticulum of >5 cm and a KD orifice diameter of >3 cm are considered thresholds for surgical intervention to avoid aortic rupture and dissection.^2^ Surgical repair is also indicated to relieve symptoms associated with vascular ring compression of the trachea and esophagus.^1^

There are several techniques for KD repair. Conventional open repair consists of replacing the involved aorta using left heart bypass or cardiopulmonary bypass and deep hypothermic circulatory arrest.^3,4^ Recently, less invasive thoracic endovascular aortic repair (TEVAR) has also been used for KD repair^2,5^; however, TEVAR is not suitable when the aortic arch is steep.

This report describes a two-stage hybrid repair involving total arch replacement followed by TEVAR, in a patient with KD, right-sided aortic arch, and aberrant right vertebral artery (RVA) to exclude KD and completely reconstruct the five cervical arteries, including the anomalous RVA, an extremely rare variant.

## Case report

**Figure 1:**
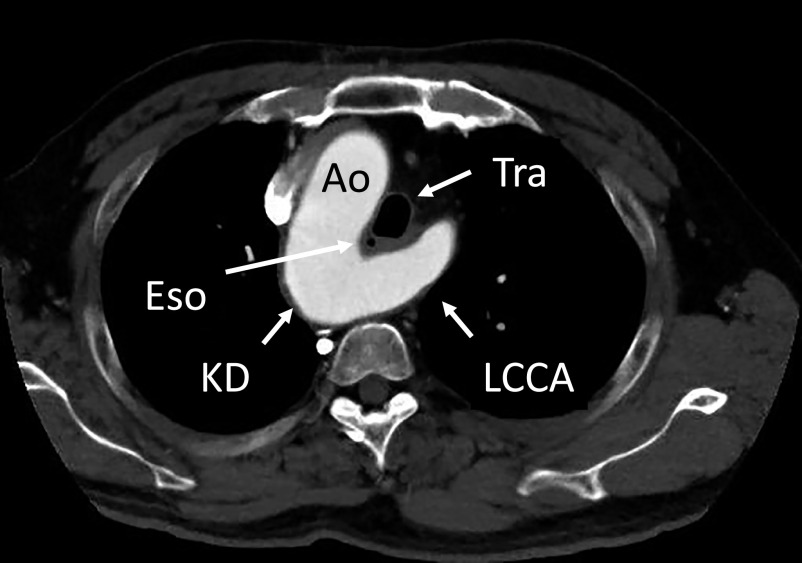
Axial computed tomography angiography showing a 26-mm aneurysmal Kommerell diverticulum (KD) with right aortic arch (Ao). The esophagus (Eso) is compressed by the trachea (Tra), and the KD connects to the common carotid artery (LCCA).

**Figure 2:**
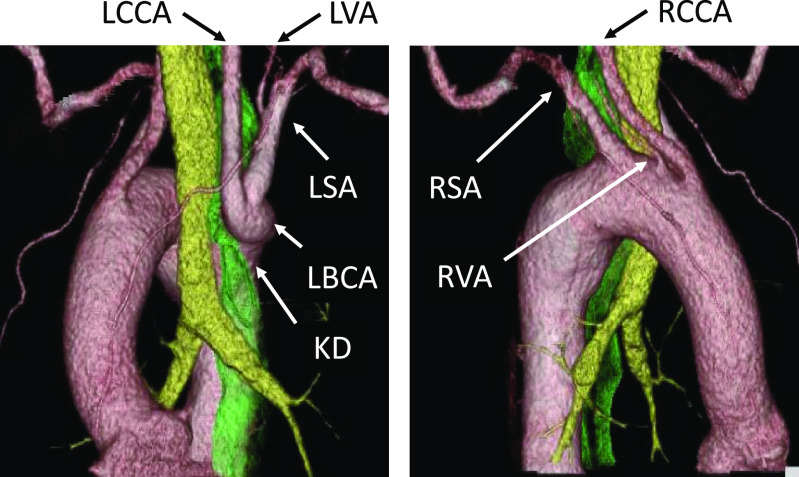
Three-dimensional computed tomography showing the right-side aortic arch branches from proximal to distal in the following order: right common carotid artery (RCCA), aberrant right vertebral artery (RVA), right subclavian artery (RSA), and the aneurysmal Kommerell diverticulum (KD). The KD connects to the left common carotid artery (LCCA), branching into the common carotid artery (LCCA) and the left subclavian artery (LSA). The esophagus (dark green) is compressed by the trachea (light green), and the KD connects to the LCCA.

A 63-year-old man presented for evaluation of dysphagia. He had previous diagnoses of infantile asthma, esophageal anomaly, duodenal ulcer, and spinal canal stenosis. Axial computed tomography angiography (Fig. 1) demonstrated a right-sided aortic arch and a 26-mm aneurysmal KD. The esophagus was compressed by the trachea, and the KD connected to the left brachiocephalic trunk (LBCT), branching to the left common carotid artery (LCCA) and left subclavian artery (LSA). Computed tomography (CT) volume-rendered reconstruction (Fig. 2) revealed that the right-side aortic arch branched from proximal to distal in the following order: right common carotid artery (RCCA), aberrant RVA, right subclavian artery (RSA), and the aneurysmal KD. According to these image findings, we diagnosed right aortic arch Stewart–Edwards type IV accompanied by KD. Surgical intervention was chosen due to the symptoms caused by the vascular ring, which were exacerbating the dysphagia and dyspnea, although the diameter of the KD (cross-sectional diameter from the opposite aortic wall to the tip of the diverticulum) was less than 50 mm. The patient’s preoperative body surface area (BSA) was 1.67 (height=163 cm and weight=62 kg), and the preoperative risk factors were hypertension and hyperlipidemia.

**Figure 3:**
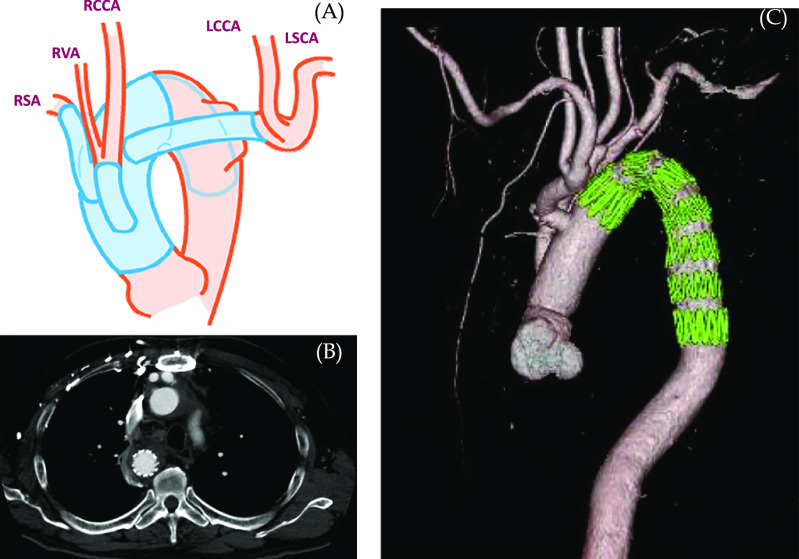
Schema (A) of the surgery. Total arch replacement with elephant trunk was performed to completely reconstruct the five cervical arteries, namely the right subclavian artery (RSA), aberrant right vertebral artery (RVA), right common carotid artery (RCCA), and left brachiocephalic trunk branching to the left common carotid artery (LCCA) and left subclavian artery (LSA). Total arch replacement was followed by second-stage TEVAR under local anesthesia, with a 36–32-mm×15.7-cm endovascular graft placed from inside the elephant trunk. Postoperative axial computed tomography angiography (B) and its three-dimensional reconstruction (C) showing successful exclusion of the KD and total arch reconstruction.

The surgical intervention involved total arch replacement with elephant trunk performed via a median sternotomy to completely reconstruct the five cervical arteries and to create an adequate landing zone, followed by second-stage TEVAR (Fig. 3). The patient first underwent total arch replacement in the supine position under general anesthesia. The left axillary and right femoral arteries were exposed for arterial cannulation. Cardiopulmonary bypass was established via the arterial cannula and right atrial drainage. The patient was cooled to 23°C, followed by lower-body circulatory arrest with deep hypothermia. Antegrade selective cerebral perfusion was established by axillary perfusion with a clamped LBCT and direct cannulations of the RCCA and RSA. The aorta was transected just distal to the origin of the RSA. A 22-mm Triplex prosthetic graft (Terumo, Tokyo, Japan) was inserted 8 cm into the descending aorta as an elephant trunk, and distal reconstruction of the aortic arch was performed with a 22-mm Triplex four-branch graft. The origin of the LBCT on the wall of the descending aorta was closed with running sutures. The RCCA and aberrant RVA, which were transected as one unit, and the RSA and LBCT (LCCA and LSA) were sequentially connected to three branches of the four-branch graft using end-to-end anastomosis; thereby, replacing the entire aortic arch with artificial vessels. The cardiopulmonary bypass time was 241 min, cardiac arrest time was 177 min, and the circulatory arrest duration of the lower body was 104 min.

Although the patient developed postoperative right recurrent nerve palsy, he could swallow and ingest food normally after rehabilitation. After confirming no abnormal findings on the CT images, he was discharged 27 days after surgery, ambulating independently.

Two weeks after discharge, the patient underwent the second surgery (TEVAR) under local anesthesia, with a Zenith TX2 TAA Endovascular Graft (Cook, Bloomington, IN) (36–32-mm×15.7-cm) placed from inside the elephant trunk. Contrast CT confirmed no obvious endoleaks (Fig. 3), and the patient was discharged 6 days after TEVAR.

During the 4-year postoperative outpatient follow-up, the patient remained healthy without symptoms of dysphagia and dyspnea and with no sign of endoleaks on CT examinations.

## Discussion

The right and left vertebral arteries normally originate as the first branches of the subclavian arteries on the respective sides. In normal anatomy, the RSA arises from fusion between the primitive fourth branchial arch and the C7 intersegmental artery. The vertebral arteries develop from a longitudinal anastomosis between C1 and C7 intersegmental arteries, followed by physiological obliteration of the right dorsal aortic arch distal to the C7 intersegmental artery.^6^ The commonest variant is a left vertebral artery arising directly from the aortic arch between the LCCA and LSA.^7^ Anomalous origins of the RVA are less frequently encountered, the commonest being an origin from the RCCA, with a concomitant anomalous RSA.^8^ In our patient, the RVA arose as the last branch of the aortic arch distal to the LSA, which is rarely associated with KD.

Surgical procedures for KD repair depend on the related anatomical features because various approaches and adjunctive methods have been reported.^1–5^ Conventionally, graft replacement from the distal arch to the descending aorta, including the KD, via posterior lateral thoracotomy at the aortic arch side or total arch replacement via a median sternotomy has been performed under circulatory arrest. However, these procedures are so invasive that there are concerns regarding high mortality and morbidity, in addition to the risks of insufficient KD excision and the requirement for additional treatment. Minimally invasive TEVAR has been increasingly performed for KD repair. However, even TEVAR with coverage of the KD requires LSA occlusion or extra-anatomic carotid–subclavian/axillary artery bypass or transposition. There is also concern that the aortic arch, especially a right aortic arch, with KD is so steep that TEVAR potentially involves the risks of inadequate length of the proximal landing zone, “bird-beak configuration”,^9^ and fracture of the bare spring of the endograft.^10^ Therefore, a hybrid procedure combining open surgery and TEVAR may be the most effective, as presented in the current report.

## Conclusion

We performed hybrid arch repair for KD with a right aortic arch and aberrant RVA as total arch replacement with elephant trunk and reconstruction of the five cervical arteries. This was followed by second-stage TEVAR from the ascending aorta to the proximal descending aorta, to exclude the KD.
